# Effect of Aerobic Exercise on Cognition, Academic Achievement, and Psychosocial Function in Children: A Systematic Review of Randomized Control Trials

**DOI:** 10.5888/pcd10.130010

**Published:** 2013-10-24

**Authors:** Caitlin Lees, Jessica Hopkins

**Affiliations:** Author Affiliations: Caitlin Lees, Dalhousie University, Halifax, Nova Scotia. At the time of this study, Dr Lees was a student at McMaster University, Hamilton, Ontario. Dr Hopkins is an assistant professor of Clinical Epidemiology and Biostatistics, McMaster University, Hamilton, Ontario.

## Abstract

**Introduction:**

Although the effects of aerobic physical activity (APA) on children’s physical health is well characterized, the effect of aerobic physical activity on cognition, academic achievement, and psychosocial function has not yet been established. This systematic review provides an overview of research elucidating the relationship between aerobic physical activity and children’s cognition, academic achievement, and psychosocial function.

**Methods:**

A systematic review of English articles was performed in April 2013 using MEDLINE, Cochrane, PsycINFO, SPORTDiscus, and EMBASE. Additional studies were identified through back-searching bibliographies. Only randomized control trials with an intervention of aerobic physical activity in children younger than 19 years that measured psychological, behavioral, cognitive, or academic outcomes were included.

**Results:**

We found 8 relevant randomized control trials that met our inclusion criteria and extracted relevant data and evaluated the methodologic quality of the studies. Of the 8 studies identified, 2 studies were crossover randomized control trials studying the effects of acute aerobic physical activity on cognitive performance. Six studies were parallel-group randomized control studies, of which only 2 had a follow-up period of longer than 6 months. All studies showed that APA had a generally positive impact on children’s cognition and psychosocial function. However, this relationship was found to be minimal in many studies and in some measures, no significant improvement was seen at all. There was no documentation of APA having any negative impact on children’s cognition and psychosocial health, even in cases where school curriculum time was reassigned from classroom teaching to aerobic physical activity.

**Conclusion:**

APA is positively associated with cognition, academic achievement, behavior, and psychosocial functioning outcomes. More rigorous trials with adequate sample sizes assessing the impact of APA on children’s cognitive abilities, psychosocial functioning, behavior, and academic achievement are needed, with standardized interventions, valid and reliable tools of measurement, and long-term follow-up for sustained cognitive and psychosocial outcomes.

## Introduction

The global trend of rising childhood obesity has become increasingly prominent; 21.4% of youth aged 5 to 17 years in Organization for Economic Co-operation and Development (OECD) countries were considered overweight or obese on the basis of 2011 data (1). This proportion represents a significant increase from previous generations. For example, in Canada the percentage of youth aged 5 to 17 years considered overweight or obese from 2009 through 2011 was 31.5%, more than double the 15% of youth aged 0 to 17 years in 1978 and 1979 (2). Along with poor diet and an obesogenic environment, inadequate physical activity is considered a key factor (3,4). On average, only 20% of children in OECD countries participate in moderate to vigorous physical activity daily (5). One study of The Canadian Health Measures Survey, which used accelerometers to objectively monitor physical activity, found that only 7% of children (a nationally representative sample aged 6–19 y) engaged in 60 minutes of moderate to vigorous physical activity daily at least 6 days per week, based on Canadian Physical Activity Guidelines (6,7).

The benefits of physical activity on health outcomes have been documented (8,9). However, the cognitive and psychosocial effects of aerobic physical activity (APA) on children are not well understood. Rasberry et al conducted a systematic review on the relationship between school-based physical activity and academic performance (10). Their review, although comprehensive, included many cross-sectional studies and studies that did not specify the type (aerobic vs non-aerobic) or dose (duration, frequency) of physical activity, making it challenging to infer optimal conditions for physical activity to enhance academic performance. Singh et al also conducted a systematic review, which focused on the longitudinal relationship between physical activity and academic performance using only prospective data (11). Although the positive association between physical activity and performance at school in longitudinal studies is supportive of a causal effect, many studies did not qualify the type or dose of physical activity. This omission is relevant, given the evidence that aerobic-based physical activity generates structural changes in the brain, such as neurogenesis, angiogenesis, increased hippocampal volume, and connectivity (12,13). In children, a positive relationship between aerobic fitness, hippocampal volume, and memory has been found (12,13). Animal and human studies have demonstrated that regular APA induces physiologic adaptations, such as an increase in blood volume, improved fat mobilization, and thermoregulation (13). More recently, the effect of APA on the brain has been studied in detail. The brain also demonstrates structural changes in response to regular APA (12,13). In particular, structural changes to the hippocampus have been observed that have implications for memory and stress regulation (12,13). Neurogenesis and increased white matter connectivity have also been observed in some studies in response to APA (13). Physiologic changes resulting from APA suggest an adaptive plasticity that could be harnessed to improve physical fitness, cognition, academic achievement, and psychosocial function. Given this relationship and the need to build on previous reviews with more specific information on the physical activity intervention, we focused on APA interventions and their effect on children’s cognitive function, academic achievement, and psychosocial function, with consideration of methodologic quality.

## Methods

### Data sources

In April 2013, we performed a computerized search of 5 large electronic databases — MEDLINE, SPORTDiscus, EMBASE, Cochrane, and PsycINFO — including results from all years available. Two elements were used in the search strategy, the first being for APA (eg, physical activity, aerobic exercise, or cardiovascular health) and the second being measures of cognitive and psychological outcomes (eg, health, mental health, cognition, achievement, intelligence tests, intelligence). The specific search terms used varied slightly in each database so as to make use of MeSH terms or subheadings. More specific keywords such as “aerobic” were at times subsumed by these headings and thus not included, and search terms were kept purposefully broad to ensure that relevant results were not excluded. Population was limited to children (younger than 19 years) either through the search limitations available or through keywords (eg, children, teens, child, students). Studies were limited to English (Appendix).

### Definitions

We used the Centers for Disease Control and Prevention’s Physical Activity Glossary of Terms (14). APA, which is intended to improve cardiorespiratory fitness, can be defined as “activity in which the body’s large muscles move in a rhythmic manner for a sustained period of time” (14). Exercise is considered a “subcategory of physical activity that is planned, structured, repetitive, and purposive in the sense that the improvement or maintenance of one or more components of physical fitness is the objective” (14). Physical activity is “any bodily movement produced by the contraction of skeletal muscle that increases energy expenditure above a basal level” (14). This differs from physical fitness, which is “the ability to carry out daily tasks with vigor and alertness, without undue fatigue, and with ample energy to enjoy leisure-time pursuits and respond to emergencies” (14).

Outcomes were grouped in the categories of mental health, behavior, and cognition. The World Health Organization defines mental health as not merely the absence of disease but “a state of well-being in which every individual realizes his or her own potential, can cope with the normal stresses of life, can work productively and fruitfully, and is able to make a contribution to her or his community” (15). We looked for studies with outcomes related to mental health disorders, such as mood disorders (eg, depression, anxiety) or attention deficit hyperactivity disorder. Behavior, in the context of this study, was used to refer specifically to behaviors that may promote or disrupt learning in the school setting, through actions, speech, or distraction. Lastly, cognition was defined as a student’s ability to learn through perception, reasoning, analysis, and judgment, which is commonly measured in schools through the use of objective tests, whereas academic achievement referred to a student’s performance on school-related work or tasks (eg, standardized tests, grades).

### Study selection

Studies of interest included those with a population younger than 19 years with an APA intervention. Inclusion and exclusion criteria were agreed on by both authors. Only randomized controlled trials were included to minimize biases between intervention and control groups and to support the causal role of the intervention. Outcomes were included only if they involved a measure of mental health, behavior, discipline, or cognition. Studies were excluded if the results measured only fitness or the success of health promotion (adherence to regimes and participation), or if the study measured only health outcomes specific to a disease state.

The search strategy guided the flow of articles through the review process (Figure) and returned 1,233 results. The citation lists for review articles and meta-analyses were back-searched for relevant articles that may have been missed by the search strategy, resulting in 12 potentially germane articles for a total of 1,245. After duplicates were excluded, there were 1,200 unique articles. A total of 1,113 results were immediately excluded according to our criteria. After further reading, we excluded a remaining 4 for focusing exclusively on an obese population, 16 for also being reviews or meta-analyses, 9 for having a pretest/posttest study design, 44 for having a cross-sectional study design, and 6 for various other reasons. This resulted in a total of 1,192 studies excluded.

**Figure:**
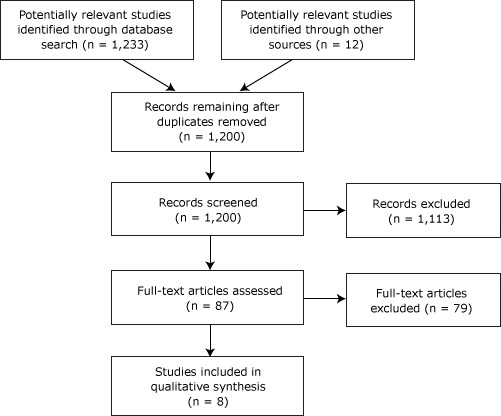
Flow of articles through the review process.

### Data extraction

Data were extracted and abstracted by a single author (C. L.). Any uncertainties regarding article categorization or data abstraction were resolved by consensus between authors. Article validity was assessed through a standardized critical appraisal tool, the Jadad Scale (16), which on systematic review was shown to have the best validity and reliability evidence of numerous scales evaluated, including the Delphi List and CONSORT Scale (17). The Jadad Scale focuses on 3 criteria that have been shown to correlate with bias: randomization, blinding, and an account of all participants. The scale ranges from 0 to 5; studies less subject to bias score higher.

## Results

We selected studies on the basis of study design, country, follow-up duration, characteristics of participants at baseline, type of APA, outcome measures, and main results (Table 1) and analyzed the studies’ strengths and weaknesses (Table 2). Although the outcomes measured were heterogeneous, all studies showed that APA had a generally positive effect on children. There was no documentation of any negative outcome resulting from APA. Shephard (24) noted that APA resulted in slight improvement in academic performance, even when curriculum time was reassigned from traditional education to physical activity. No study analyzed a dose–response relationship between APA and cognitive, mental health, or behavioral outcomes.

**Table 1:** Randomized Controlled Trials Reporting Children’s Cognitive and Psychosocial Outcomes After Increased Aerobic Activity

Source	Study Design/Country/Follow-up Duration	Characteristics of Study Sample at Baseline	Measure of APA	Measure of Outcome	Main Results
Crews et al 2004 (18)	RCT/United States/6 weeks	66 Grade 4 (aged 8–10 y) Hispanic students in a low-income school district, 33 boys and 33 girls	Aerobic physical activity with an intensity equal or greater than 60% of maximum heart rate reserve	State-Trait Anxiety Inventory (STAI), Beck Depression Inventory, Rosenberg’s Scale	Children participating in an aerobic exercise program of 20 minutes, 3 times per week, reported significantly less depression and greater self-esteem, but no difference on trait anxiety.
Donnelly et al 2009 (19)	RCT/United States/3 years	527 Grades 2 and 3 students (aged 6–9 y)	45 or More minutes per week of moderate to vigorous intensity physical activity, measured by accelerometry	Wechsler Individual Achievement Test, 2nd edition (WIAT-II)	Significant improvements in academic achievement from baseline to 3 years of follow-up were seen in schools with aerobic exercise programs.
Fisher et al 2011 (20)	RCT/Scotland/10 weeks	64 Year 2 children (aged 5–7 y), 33 boys and 31 girls	2 Hours of aerobically focused physical education (PE) per week, measured by accelerometry	Cognitive Assessment System (CAS), Cambridge Neuropsychological Test Battery (CANTAB), Attention Network Test (ANT), Conner’s Behavior Rating Scale	Children engaging in aerobically focused PE showed no significant difference in CAS scores, but CANTAB spatial span and spatial working memory errors as well as ANT accuracy scores were improved in the intervention group.
Hill et al 2011 (21)	Crossover/Scotland/2 weeks	760 Children in primaries 4 to 7 (aged 8–12 y)	On each cohorts’ scheduled week, 10–15 minutes of moderately intensive APA daily	Cognitive test battery	APA had a short-term positive effect on cognitive performance, but may have been due to exercise enhancing use of memory (practice).
Reed et al 2010 (22)	RCT/United States/4 months	155 Children in 3rd grade (aged 7–10 y)	30 Minutes of integrated APA into core curriculum,3 times per week, with pedometers to measure activity. Evidence supports walking as moderate-intensity APA (23).	Palmetto Achievement Challenge Tests (PACT), Standard Progressive Matrices (SPM), Fluid Intelligence Tests	Children performing regular integrated aerobic activity performed significantly better on intelligence testing and on state tests on social studies. Scores were also higher on English/language arts, math, and science achievement tests but not significant in comparison to control groups.
Shephard 1996 (24)	RCT/Canada/6 years	546 Children from grades 1 through 6 (aged 5–12 y)	Additional 1 hour of aerobic physical activity daily, with heart rates between 157 and 178 beats per minute	Goodenough test, Wechsler Intelligence Scale for Children (WISC)	APA enhances (rather than worsens) academic performance even if curriculum time is reassigned to physical activity; however, effects are very small.
Stroth et al 2009 (25)	Crossover/Germany/7 days	33 Adolescents (aged 12–16 y), divided into higher-fit (n = 17) and lower-fit (n = 16) subgroups	Acute bout (20 min) of moderate APA, corresponding to 60% of individual maximal heart rate	Modified Eriksen flanker task, evaluating executive function through go/nogo tasks	No reliable effect of acute exercise on inattention (missed responses to “go-trials”) or impulsivity (false alarms in nogo-trials); however, higher-fit individuals did perform better.
Tuckman and Hinkle 1986 (26)	RCT/United States/12 weeks	154 Children in grades 4, 5, and 6 (mean ages 9.3, 10.3, and 11.3, respectively)	Three sessions of running for 30 minutes each week	Devereux Elementary School Behavior Rating Scale, Alternate Uses Test, Maze Tracing Speed Test	Running (aerobic exercise) had a significant positive effect on creative capacity but made no contribution to behavior or perceptual functioning.

Abbreviations: APA, aerobic physical activity; RCT, randomized controlled trial.

**Table 2:** Critical Appraisal of Randomized Control Studies Reporting Children’s Cognitive and Psychosocial Outcomes After Increased Aerobic Activity

Source	Study Strengths	Study Weaknesses	Critical Appraisal: Jadad Criteria (range, 0–5) (16)
Crews et al 2004 (18)	Standardized measure of APA and outcome; measures used were common and validated (27,28); high internal reliability of inventories (test retest, α = 0.90)	Small number of participants, short follow-up; Beck Depression Inventory version was not designed for children; STAI-Y has not been validated in children	1
Donnelly et al 2009 (19)	Large number of participants; long follow-up; standardized measure of APA and outcome; WIAT-II validated, reliable, and commonly used in children (29)	None	2
Fisher et al 2011 (20)	Standardized measure of APA and outcome; CAS previously demonstrated to be both valid and reliable in children older than 4 years with high correlation to IQ testing (29); Conner’s Parent Rating Scale validated and reliable in children (30)	Small number of participants; short follow-up; Cambridge Neuropsychological Test Battery (CANTAB) and Attention Network Test (ANT), Conner’s Parent Rating Scale not validated in children (reliability testing by authors found limited utility with poor to moderate reliability); fewer than half the parents returned the Conner’s questionnaire	4
Hill et al 2011 (21)	Large number of participants; objective measure of outcome	Short follow-up; no objective measure of APA; cognitive battery test used modified versions of the Children’s Size Ordering Task, Wechsler Intelligence Scale for Children (WISC), and Children’s Paced Auditory Serial Addition Task, so validity and reliability cannot be judged	4
Reed et al 2010 (22)	Standardized measure of outcome; SPM has high reliability and is recommended for children under 12 years (29)	Short follow-up; did not measure intensity of APA; Palmetto Achievement Challenge Tests (PACT) not validated based on scholarly database search or cited in the article	4
Shephard 1996 (24)	Large number of participants; long follow-up; standardized measure of APA and outcome; WISC is a commonly used measure of global intelligence in children and is both reliable and valid (28)	Goodenough test shows moderate correlation to intelligence but is best corroborated with other assessments (28)	0
Stroth et al 2009 (25)	Standardized measure of APA and outcomes	Small number of participants; short follow-up; go/nogo test only used and previously validated for use in neurophysiology testing by the authors but not for the results indicative of executive function, so reliability and validity in this context cannot be judged (31)	2
Tuckman and Hinkle 1986 (26)	Standardized measure of outcome; Devereux scale and Alternate Uses Test reliable	Short follow-up; no standardized measure of APA; Maze Tracing Speed Test has no reliability information	1

Abbreviations: APA, aerobic physical activity; STAI-Y, State-Trait Anxiety Inventory Form Y; WIAT-II, Wechsler Individual Achievement Test, 2nd edition; CAS, Cognitive Assessment System; IQ, intelligence quotient; SPM, Standard Progressive Matrices Test; WISC, Wechsler Intelligence Scale for Children.

Mental health outcomes included reduced depression and increased self-esteem, although no change was found in anxiety levels (18). Tuckman et al found a positive effect on creative capacity but no change in behavior or perceptual functioning (26). Similarly, Stroth et al found no consistent effect of acute APA on inattention or impulsivity; however, they did note that subjects who were already more fit (and ostensibly participated in APA more frequently) did perform better (25).

All 4 studies that measured academic achievement or cognitive performance noted enhanced performance in the APA intervention group. One of the longest running studies measured academic performance over 6 years and found a small improvement in academic performance in children from grades 1 through 6 (24). Hill and colleagues’ (21) short crossover trial showed short-term improvement on cognitive performance, but the authors admitted that their research methods may have had this effect because APA enhanced participants’ use of memory from practice. Donnelly et al (19) found significant improvements in academic achievement over 3 years of follow-up in schools with APA programs, and Reed et al (22) found that children participating in APA, even engaging only in moderate-intensity APA as measured by pedometers (23), performed significantly better on intelligence testing and state tests on social studies. Scores were noted to be higher on English/language arts, math, and science tests, but the results were not significant.

## Discussion

This systematic review of the literature found that APA is positively associated with cognition, academic achievement, behavior, and psychosocial functioning outcomes. Importantly, Shephard also showed that curriculum time reassigned to APA still results in a measurable, albeit small, improvement in academic performance (24). This finding lends support to the theories that APA and curricular time need not be a trade-off, that there is a synergistic effect between APA and academic performance, and that educators and policy makers can be reassured that spending time in APA does not detract from academic achievement.

Strengths of this review included its systematic approach and thorough, replicable search strategy. This study builds on previous reviews by using methodologically stronger study designs that support causal hypotheses and standardized measures of APA and outcomes. We excluded studies that examined specific populations, notably overweight or obese children, in an attempt to reduce potentially confounding factors and to allow for generalizability at a population level. Although studies that used self-reported academic achievement or cognitive scores were not part of the original inclusion/exclusion criteria, no studies were included that did, which significantly improves validity.

This systematic review was limited by the available studies. This study did not include either unpublished research findings or non-English studies, which may have resulted in the loss of relevant research. Publication bias may also have resulted in relevant studies (especially those demonstrating an equivocal outcome) not being published. Although the similar outcomes of cognition, academic achievement, and psychosocial functioning were measured in all of the studies, measurement tools were inconsistent between studies, and some lacked validity data (Table 2). Academic achievement does not necessarily correlate with cognitive ability, although presumably there may be some overlap in areas of perception, reasoning, analysis, and judgment. In some cases, studies were also limited by small participant numbers (underpowered) and short follow-up times. Additionally, only 1 study explored mental health–related outcomes (18).

Although this review cannot provide a definitive answer to the question of whether APA leads to improved cognition, academic achievement, behavior, and psychosocial functioning, the results of a positive association are promising and worthy of future, more rigorous study. APA interventions in a school-based setting are complex to administer. A perfectly designed randomized control trial may be limited by generalizability to other settings. By using the information obtained from a diverse set of interventions and outcome measures, some interesting patterns emerge. The actual aerobic-based activity does not appear to be a major factor; interventions used many different types of APA and found similar associations. In positive association studies, intensity of the aerobic activity was moderate to vigorous. The amount of time spent in APA varied significantly between studies; however, even as little as 45 minutes per week appeared to have a benefit. 

Although all of these areas require further study, our findings can inform educators and policy makers as they work to justify, support, and integrate APA into the regular school curriculum. Future study should focus on the dose–response relationship between APA and cognitive or psychosocial outcomes and the optimum frequency of bouts of APA, neither of which has been adequately investigated. More rigorous trials with adequate sample sizes assessing the effect of APA on children’s cognitive abilities, psychosocial functioning, behavior, and academic achievement are needed, with standardized interventions, valid and reliable tools of measurement, and long-term follow-up for sustained cognitive and psychosocial outcomes.

## Appendix: Search Strategies

MEDLINE

- (Physical Fitness OR Exercise OR Physical Exertion) AND (Mental Health OR Health OR Cognition OR Achievement OR Intelligence)
- Subject Headings used
- Limited to English language, Human, All Children (0-18 y)
- 479 hits

SPORTDiscus

- (Physical Activity OR Exercise OR Cardiovascular OR Aerobic) AND (Health OR Mental Health OR Cognition OR Achievement OR Intelligence OR Intelligence Tests) AND (Children OR Teens OR Child OR Students)
- Keyword search
- Limited to English language
- 54 hits

EMBASE

- (Physical Activity OR Exercise OR Aerobic OR Fitness) AND (Achievement OR Cognition OR Health OR Intelligence OR Mental Health)
- Subject Headings used
- Limited to Human, English language, and Child (unspecified age). MEDLINE journals excluded.
- 58 hits

Cochrane

- (Exercise OR Physical Fitness OR Aerobic OR Physical Exertion) AND (Cognition OR Mental Health OR Achievement OR Intelligence OR Intelligence Tests)
- Keywords used
- 3 hits

PsycINFO

- (Physical Activity OR Exercise OR Fitness) AND (Mental Health OR Health OR Cognition OR Intelligence OR Intelligence Test)
- Major Subject Headings used
- Limited to Childhood (birth–12 y), Adolescence (13–17 y), School Age (6–12 y) and English language
- 639 hits
